# Dissociating Attention and Eye Movements in a Quantitative Analysis of Attention Allocation

**DOI:** 10.3389/fpsyg.2017.00715

**Published:** 2017-05-17

**Authors:** Gene M. Heyman, Jaime Montemayor, Katherine A. Grisanzio

**Affiliations:** ^1^Department of Psychology, Boston College, Chestnut HillBoston, MA, USA; ^2^Department of Biomedical Sciences, Tufts University School of MedicineBoston, MA, USA; ^3^Department of Psychiatry and Behavioral Sciences, Stanford University School of MedicineStanford, CA, USA

**Keywords:** covert attention, overt attention, attention allocation, choice, eye movements, fixations, eye-tracking, mathematical model of attention allocation

## Abstract

In a recent paper, we introduced a method and equation for inferring the allocation of attention on a continuous scale. The size of the stimuli, the estimated size of the fovea, and the pattern of results implied that the subjects' responses reflected shifts in covert attention rather than shifts in eye movements. This report describes an experiment that tests this implication. We measured eye movements. The monitor briefly displayed (e.g., 130 ms) two small stimuli (≈1.0° × 1.2°), situated one atop another. When the stimuli were close together, as in the previous study, fixations that supported correct responses at one stimulus also supported correct responses at the other stimulus, as measured over the entire session. Yet, on any particular trial, correct responses were limited to just one stimulus. This pattern suggests that the constraints on responding within a trial were due to limits on cognitive processing, whereas the ability to respond correctly to either stimulus on different trials must have entailed shifts in attention (that were not accompanied by eye movements). In contrast, when the stimuli were far apart, fixations that had a high probability of supporting correct responses at one stimulus had a low probability of supporting correct responses at the other stimulus. Thus, conditions could be arranged so that correct responses depended on eye movements, whereas in the “standard” procedure, correct responses were independent of eye movements. The results dissociate covert and overt attention and support the claim that our procedure measures covert attention.

## Introduction

Fechner's *Elemente der Psychophysik*, published in 1860 has long stood as a landmark in the history of experimental psychology. Fechner believed that the workings of the mind followed simple mathematical rules, and, then, in what proved to be a step into the future, he experimentally tested his beliefs, creating psychophysics in the process. Generations of researchers have followed Fechner's lead: first specifying a quantitative model of the mind then testing it experimentally (see, e.g., signal detection theory studies). Working in this tradition, we recently introduced a procedure for quantitatively inferring the allocation of attention (Heyman et al., 2016). One of our goals has been to test whether mathematical principles that describe the allocation of overt behavior, such as maximizing reward, the matching law, and probability matching, also describe the allocation of attention under analogous conditions (Herrnstein et al., 1997; Shanks et al., 2002). In the behavioral studies, researchers routinely measure choice proportions on a continuous scale from 0 to 100%. The methodological challenge was how to quantify the allocation of attention in the same way. Although there is a long tradition of quantification in cognitive psychology (e.g., Sperling and Dosher, 1986; Bundesen, 1996), there is, to our knowledge, no method for inferring the allocation of attention on a continuous scale that ranges from 0 to 100% (as in choice studies). Our solution was an attention task that could be modeled by a linear equation. One of the unknowns was the allocation of attention. Thus, solving the equation also “solved” how much attention a subject devoted to each stimulus.

The results of the first study supported the idea that quantitative principles that describe the allocation of choice also describe the allocation of attention. Tests of the validity of the calculations and their supporting assumptions were positive and encouraging. However, one assumption regarding the validity of the equation went untested. On the basis of the size of the stimuli and brief presentation times (e.g., on average about 130 ms), we assumed that the equation measured covert attention not eye movements. This report describes the results of an experiment that tested this assumption.

According to standard texts, the foveola, located in the center of the fovea, encompasses projected images of about 1.2° (Wandell, 1995; Wolfe et al., 2009). In our study, the entire stimulus array projected an image of 0.95° × 1.17°. Thus, a fixation that captured one stimulus should have also captured the other stimulus. Yet, on any particular trial, correct responses were limited to either the top or bottom stimulus. This suggests that the limitation was due to limitations in covert processes not to insufficiently fast eye movements or other possible visual constraints. We tested this inference as follows.

We calculated the probability of a correct response as a function of where the subject was looking. Fixations were mapped onto a six-sectioned grid, referred to as “areas of interest” (AOIs). If correct responses depended on shifts in attention rather than shifts in eye movements, then it should be possible for the same AOI to support correct top and correct bottom responses on different trials. To see this, consider the following possible sequence of events. Assume the computer picks the top stimulus as correct and the subject can successfully attend to one of the stimuli but not both on any given trial because of the brief presentation times. If the subject attended the top stimulus, she responds correctly, but if she had attended the bottom stimulus, then the only way she can make a correct response is by guessing (although her eye had focused an image of the top stimulus on her fovea). Then on the next trial, assume the computer picks the bottom stimulus as correct and the subject maintains her gaze at the AOI that on the previous trial led to a correct top response but shifts her attention to the bottom stimulus: again a correct response. Thus, the same fixation, as measured by AOI, can maintain correct top stimulus and correct bottom stimulus responses, even though on any one trial it is only possible to attend to one of the two. Notice also, that under the conditions just described, a fixation that led to correct responses will yield incorrect responses if the subject attended the stimulus that did not provide the correct answer (absent correct guesses)—even though an image of that stimulus was projected onto the fovea. Thus, this procedure has the capacity to dissociate shifts in attention from shifts in eye movements.

In previous studies, researchers typically used response times to distinguish between shifts in attention and shifts in eye movements (e.g., Eriksen and Hoffman, 1973; Posner, 1980; Hunt and Kingstone, 2003), and with few exceptions did not take advantage of eye tracking to measure overt attention (e.g., de Haan et al., 2008; Amir et al., 2016). Our approach seems more direct in that it involves a record of where the subjects were looking and where they were attending. However, we should add that the procedure's capacity to dissociate covert and overt attention is a consequence of our more general goal of establishing a way to measure covert attention on a continuous scale. Before describing the results, we will introduce the model and the results which support its validity.

### A cognitive choice procedure

The procedure is a cognitive version of the much studied “two-armed bandit” procedure used in choice studies (Estes, 1976; Shettleworth and Plowright, 1989; Gaissmaier and Schooler, 2008; Kwak et al., 2014; McDougle et al., 2016). The monitor displayed two stimuli. One provided the information needed to make a correct response; the other did not. As described in the *Methods* section, exposure times were calibrated to ensure that the subject had enough time to respond correctly to one stimulus but not both. Fixed probabilities that summed to 1.0 determined which stimulus was correct. The subject could get more correct responses by learning which stimulus the probabilities favored. In analogous behavioral “two-armed bandit” procedures, subjects learn to favor the option that is more likely to payoff. However, preferences often fall short of the ratio predicted by a maximizing strategy. For instance, the maximizing strategy is to choose the more likely alternative on every trial, whereas the observed choice ratios tend to approximate the arranged probabilities of correct responses. This is called “probability matching,” and it has attracted much attention because it appears to violate the assumptions of rational choice theory (e.g., Vulkan, 2000).

However, the choice literature also shows that under some circumstances probability matching sometimes gives way to maximizing. As a function of feedback regarding correct responses, incentives, and practice, many subjects shift toward maximizing (e.g., Fantino and Esfandiari, 2002; Shanks et al., 2002). This is what we found using attention as the dependent variable. When there was feedback, attention allocation ratios deviated from probability matching toward maximizing; whereas when there was no arranged feedback, as in the study described in this report, attention allocation ratios approximated the probability matching predictions. To summarize: the independent variable is the programmed probabilities that the top and bottom stimuli are correct, and the dependent measures are the attention allocation ratios and the likelihood of a correct response as a function of a visual fixation's AOI (where the subject was looking). The predictions are that (1) attention ratios will approximate the probabilities of a correct response, that (2) when the stimuli are close together, the same AOI can support correct answers at both stimuli, which is to say attention shifted but eye movements did not, and that (3) when the stimuli are sufficiently far apart, shifts in attention will correspond to shifts in eye movements.

### An equation for inferring the allocation of attention

In this procedure, a linear equation describes the relationship between the allocation of attention and the percentage of correct responses:

(1) $$\begin{array}{lll} Expected\;\%\;of\;correct\;matches & = & PTp\;+\;PT\left(1-p\right)g\\ +\;PB\left(1-p\right)\;+\;PBpg, \end{array}$$

where *PT* is the probability that the experimenter set the top stimulus as the correct one, PB is the probability that the bottom stimulus is the correct one, *p* is the probability that the participant attended to the top row, (1-*p*) is the probability that the participant attended to the bottom row, and *g* is the frequency of correct guesses. Given these definitions, we can solve Equation 1 for *p* (the allocation of attention) and *g* (the correct guess rate). The results along with modifications that take into account non-attentional factors, such as arithmetic errors (*A*) are:

(2a) $$\begin{array}{lll} p & = & \left(PCB-A\right)/\left(PCT\;+\;PCB-2A\right) \end{array}$$

(2b) $$\begin{array}{lll} g & = & \left(PCT\;+\;PCB\right)\;-\;A, \end{array}$$

where *PCT* is the probability of a correct response when the top stimulus is correct, PCB is the probability of a correct response when the bottom stimulus is correct, and *A*, which stands for accuracy, measures the likelihood of an arithmetic error or not paying attention to either stimulus (see Heyman et al., 2016 for details). *A* is not a fitted parameter, but is based on performance in trials in which the subject was told beforehand which stimulus is correct. These are referred to as “cued” trials. We included them as a way of determining how much time the subjects needed to make a correct response when they attended the correct stimulus (details below).

### Results, supporting data, and proof of concept

To test whether the equation provided valid measures of attention we evaluated two corollaries of Equation 1.

#### Correct guess rate

Each trial ended with a screen that displayed seven possible correct answers. If the subjects had attended the incorrect stimulus and had no usable information regarding the correct stimulus, the correct guess rate, represented by *g* in Equations 1 and 2b, should converge to a value of 0.143. We tested this prediction by fitting Equation 2b to the probabilities of a correct response for each of the 102 subjects that participated in the first study. The average value of *g* (0.149) differed little from the expected value (0.143). We rely on this measure in the present study.

#### Response latencies

Although Equation 1 does not directly address response times, it is reasonable to suppose that the latencies for correct responses will differ as a function of the degree to which the subject has learned which stimulus is more likely to be the correct one. The response times changed in the predicted ways as a function of experience. Response times at the more predictive stimulus on uncued trials approached those obtained in trials in which the subject was told beforehand which stimulus to attend, whereas response times at the less predictive stimulus increased, eventually exceeding those of the more predictive stimulus by about 1500 ms (see **Figure 4**, Heyman et al., 2016). We did not report response times in the present experiment.

#### Hypothesis

Our hypothesis is that when the duration of the stimuli is set so that the subject can respond correctly to only one stimulus, and the distance between the stimuli approximates the distance in the initial study, she will, nevertheless, be able to respond correctly to either one on different trials without shifting her gaze. However, to insure that we could detect a correlation between eye movements and correct responses, we explored how far apart the stimuli needed to be in order for a fixation to support correct responses at just the top stimulus or at just the bottom stimulus. On the basis of pilot sessions, we settled on a distance of 15°. Thus, the study tests the relationship between visual distance and the probability of a correct response. Half of the subjects were in the standard, 0.32° condition, and half were in the 15° condition. The pilot trials also determined the number of subjects we tested and how we aggregated the data, as described below.

## Materials and methods

### Participants

We tested 24 subjects. The median age was 19, with a range of 18 to 21. Sixteen were females. Prior to the start of the experiment all participants signed a consent form according to the protocols established by the Boston College institutional review board for research. In addition, subjects filled out a form that included questions regarding age, gender, year in school, and other demographic characteristics. The subjects earned course credit for participating in the study. The procedure was approved by the Boston College institutional review board.

### Equipment

The monitor that displayed experimental events measured 33.7 × 27.3 cm and was set at a resolution of 1,280 × 1,024 pixels. Eye movements were tracked by a SMI iView X Hi-Speed 1,250 eye tracker, set to a recording rate of 500 Hz, with attached chin and headrest to help stabilize viewing. This system provided a 2 ms continuous history of saccades, blinks, and fixations on the basis of infrared illuminated corneal reflections. A keyboard that was placed below the chin and headrest was used to record the subject's responses. E-Prime software controlled experimental events.

### Procedure

**Figure 1:**
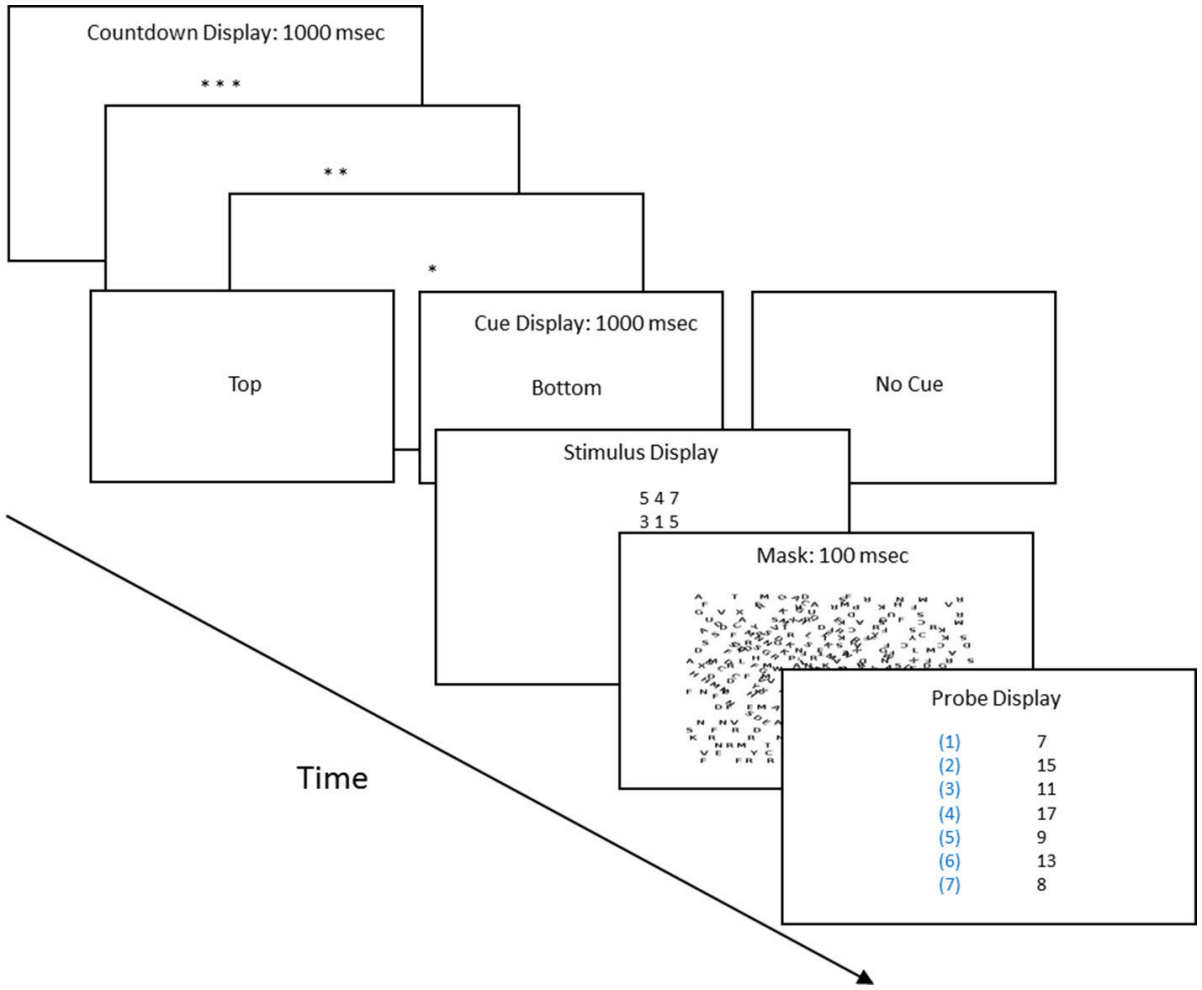
**Procedure flow chart: the procedure proceeded in four steps**. (1) Preparatory count down screens, with each asterisk screen displayed for 1 s. (2) Type of trial screen, which indicated whether it was a cued or uncued trial, and if cued, whether the top or bottom row of the stimulus held the three digits whose sum matched one of the sums in the probe screen. (3) The stimulus screen, whose duration was determined by the calibration procedure, and (4) The probe screen, which listed seven sums; one of which matched the sum of either the three digits of the top row or bottom row of the stimulus screen.

Each subject in the 0.32° and 15° conditions completed two sessions at one of the four probability conditions (16 subjects, as described below). Each session began with a calibration procedure for determining the duration of the stimulus screen. There were two types of calibration trials. On cued trials, the preparatory countdown period (the asterisk screens, see Figure 1) ended with the words “top” or “bottom,” indicating which stimulus was correct. On the not-cued trials there was a 50:50 chance the top or bottom was correct. Our goal was to find the duration that supported correct responses on at least 85% of the trials in which the subject knew beforehand which stimulus was correct (cued trials), but yielded chance performance when there was no advanced knowledge and the top and bottom stimuli were equally informative (not-cued, 50:50 trials). In the calibration session, three-quarters of the trials were cued. In the experiment proper 22 trials were cued and 110 trials were not cued, but as described, each had a fixed, learnable probability of being correct. We included cued trials in the experiment to check whether the calibration procedure had resulted in a sufficiently long exposure period for the subjects to correctly respond once they had learned which stimulus was most likely to be correct.

Figure 1 outlines the structure of each trial. The key steps were the stimulus and probe screens. The stimulus screen displayed six digits, arranged in two rows of three each. The probe screen listed seven numbers. One was equal to the sum of the three digits of the top row or to the sum of the three digits of the bottom row of the stimulus screen. Each of these sums was identified by a numerical label, as shown in Figure 1. The subject's task was to add up the three digits of the top or bottom row of the stimulus screen and see if the sum was listed on the probe screen. If so the subject pressed the key that had its corresponding label.

Whether the top or bottom row of digits had a matching sum in the probe screen was determined by fixed probabilities, which added to 1.0. For each subject, these remained fixed at one of four possible settings: 0.10, 0.90, 0.25, and 0.75. Thus, as a function of experience, the subjects could learn whether the top or bottom stimulus was more likely to be correct, but they could not be correct on every trial.

### Setting and stimuli

Each row of 3 digits of the stimulus screen measured approximately 0.89 by 0.32 cm. In the standard procedure, a 0.30 cm gap separated the two stimuli; in the 15°Condition, a 14.22 cm gap separated the two stimuli. The 0.30 cm gap is slightly larger than the gap in the initial study (0.24 cm), which reflects differences in the monitors. In angular degrees, each row of three digits measured approximately 0.94 × 0.34° and was separated by a 0.32° gap in the standard condition.

### Eye-tracking

**Figure 2:**
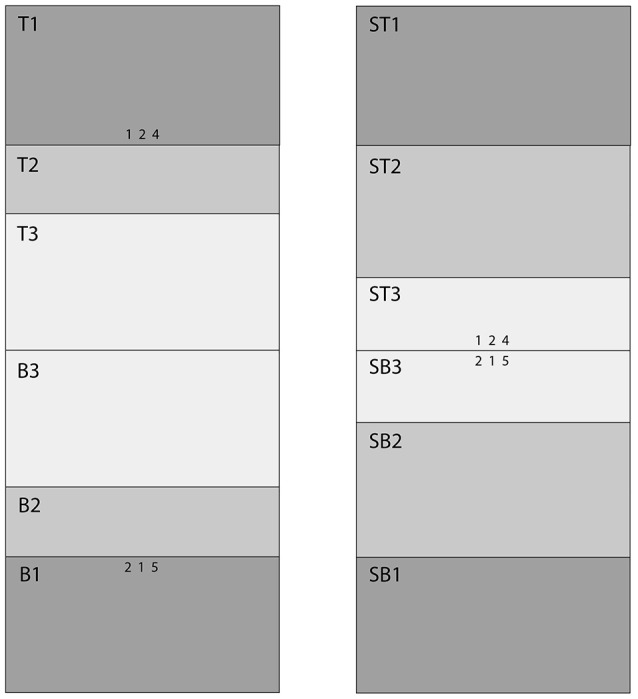
**AOI Map**. The left panel shows the AOIs for the 15°condition. The right panel shows the AOIs for the 0.32° (standard) condition. The stimuli (three digit rows) and AOIs are approximately to scale.

Figure 2 shows the Areas of Interest (AOIs) for classifying fixations. Our goal was to estimate the probability of a correct response as a function of a fixation's AOI. Participants sat 54 cm from the monitor. Prior to each session, we adjusted the eye-tracking apparatus so that fixations were as close as possible to nine preset calibration dots. During the experimental session, the eye tracker ran continuously, producing a “gaze stream” that was analyzed with SensoMotoric Instruments' BeGaze software. BeGaze was set to “High Speed,” which distinguishes saccades, blinks, and fixations on the basis of the velocity of the gaze stream and pupil diameter. The velocity threshold for saccades was 40° a second, and the duration threshold for fixations was 100 ms. That is, a gaze had to remain in an AOI for 100 ms to count as a fixation. In the pilot sessions, we discovered that fixations began in the count-down preparatory period (see Figure 1); consequently, the fixation clock started timing during the preparatory countdown screens (the asterisks in Figure 1). We used MatLab to coordinate the BeGaze fixation history, organized by AOI, and the history of correct and incorrect responses, which were recorded by the E-Prime program.

### Data analysis and design

The primary goal of the study was to determine whether eye movements were correlated with shifts in attention in the condition that mimicked our first study (“standard,” 0.32° stimulus gap) and the 15° condition. The secondary goals were to evaluate the likelihood of a correct response as a function of a fixation's distance from the target stimulus and to test the generality of the results observed in the first study (e.g., probability matching). To answer these questions we recorded the probability of a correct response as a function of AOI and as a function of the probabilities that the top and bottom stimuli had a matching sum in the probe screen in the standard and 15° conditions.

The results from the pilot studies and our earlier study determined the number of subjects in each condition. The earlier study used 6–8 subjects for each different probability that the three digits in the top stimulus row had a matching sum in the probe screen. The pilot data indicated that the relationship between an AOI and the probability of a correct response was strong and independent of how well the subject had learned which stimulus was more likely to be correct. As noted above, in this study there were four probability conditions regarding the likelihoods of correct top and bottom stimuli. From the first 24 subjects we then selected the 2 subjects who had the smallest average deviation in the eye-tracking calibration procedure for each of four different probability settings for the standard gap condition and the 15° gap condition. Thus, there were 8 subjects in the standard condition and 8 subjects in the 15° condition.

We calculated the probabilities of a correct response for those AOIs that were “visited” at least 10 times over the course of the experiment, pooling across subjects. We pooled data in order to increase the number of AOIs that could be included in the analyses. For instance, subjects rarely visited distant AOIs (relative to the target stimuli) so that not pooling would have limited the range AOIs we could enter into the analyses. In support of this approach, the relationship between AOI and probability of a correct response was orderly across the entire range of distances (see, **Figure 7**). In addition, we calculated the Spearman rank correlation coefficients for the probabilities of correct top and bottom responses as a function of AOI. If the AOIs for correct top and bottom responses overlapped, then the rank order of the probabilities of a correct response as a function of AOI would be positively correlated, whereas if the AOIs for correct top and bottom responses differed then the rank order of the probabilities of a correct response as a function of AOI would be negatively correlated.

To avoid ambiguity we restricted the calculations to trials in which fixations were limited to one AOI. If there was more than one point of regard, it would not be possible to know which one provided the information for a correct response. In 22% of the trials there were two points of regard. To check if eliminating these trials affected the interpretation of the results, we also calculated the probability of a correct response as a function of cumulative time spent in each AOI across all trials (regardless of whether the subjects shifted their gaze to a second AOI).

Angular distance from the point of fixation to the target stimuli was defined as the distance. from the midpoint of the correct stimulus to the midpoint of the AOI (with a distance set to 0.0 for the AOI in which the stimuli were embedded in the 15° condition). This is an approximation, as is explained further in the *Discussion* section. Other details of the analyses include the following.

In sum, we evaluated the relationship between eye movements and shifts in attention by calculating the probability of a correct response as a function of AOI. We determined whether we could detect a correlation between eye movements and correct responses by including two different stimulus gaps. And we tested the generality of the earlier results by using four different probability settings for the likelihood that a stimulus had a matching sum in the probe screen.

## Results

**Figure 3:**
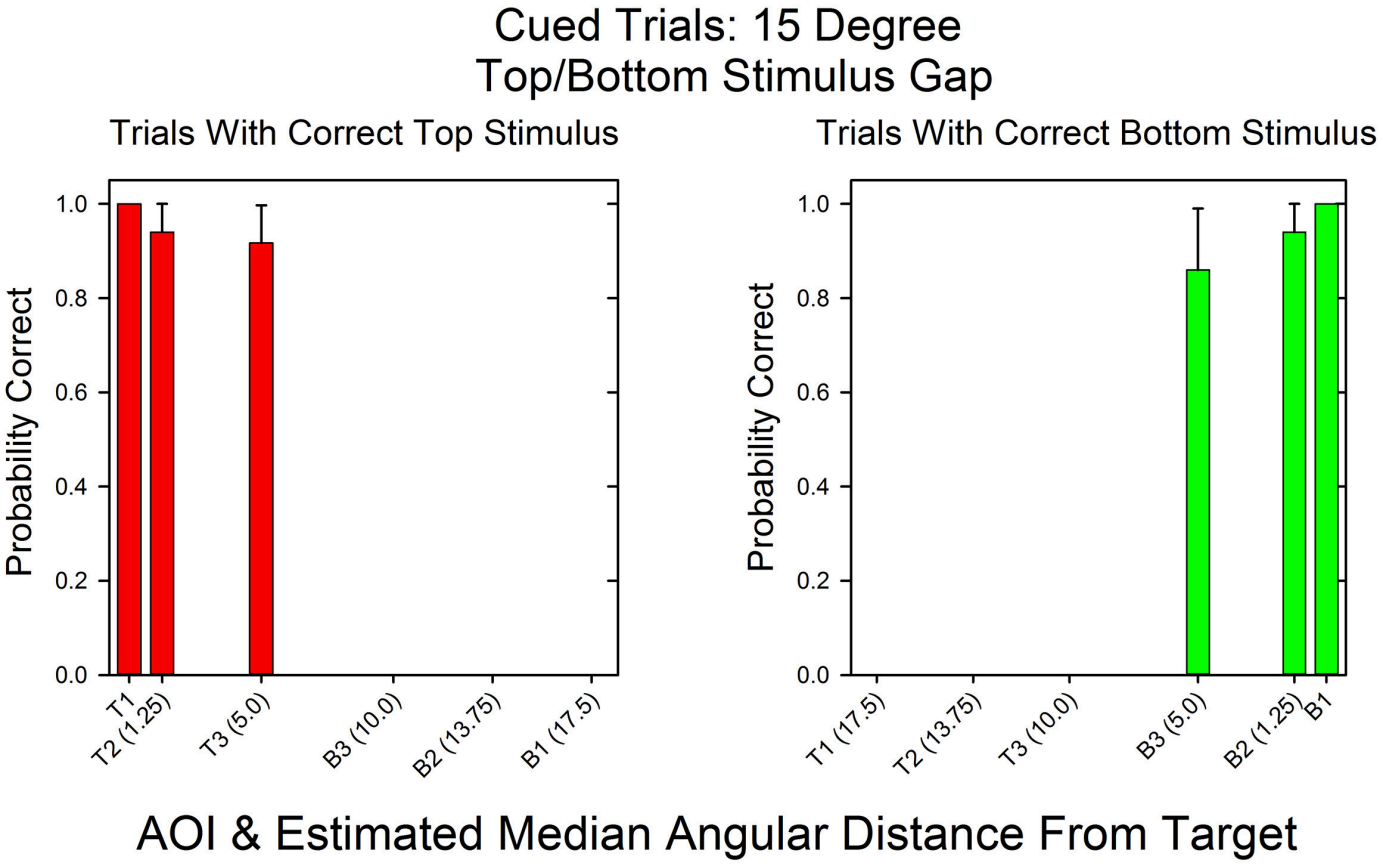
**Probability of a correct response as a function of AOI in cued trials in the 15°Condition**. On the x-axis are the AOI and its median midpoint distance from the stimulus. On the y-axis is the probability of a correct response given the fixation's AOI. The calculations are based on the pooled results across all subjects and probability conditions in the 15° stimulus gap condition.

Figure 3 shows the likelihoods of correct top stimulus and correct bottom stimulus responses as a function of AOI on cued trials in which the top and bottom stimuli were separated by 15°. In this condition, the AOIs for correct top and bottom responses should differ and there should be few if any incorrect responses due to attending to the wrong stimulus. As expected, the AOIs for correct top and bottom responses were mutually exclusive. When the top stimulus was correct, the AOIs for correct responses were *T1* to *T3* (see Figure 2). When the bottom stimulus was correct, the AOIs for correct responses were *B1* to *B3*. The left and right sides of the graph are virtual mirror images. Accordingly, the Spearman's rank correlation for the probabilities of correct top and bottom responses as a function of AOI is negative: *r*_*s*_ = −1.0, *p* < 0.05. Notice though that there were few AOIs. This is because there was no uncertainty as to where to look in order to respond correctly.

**Figure 4:**
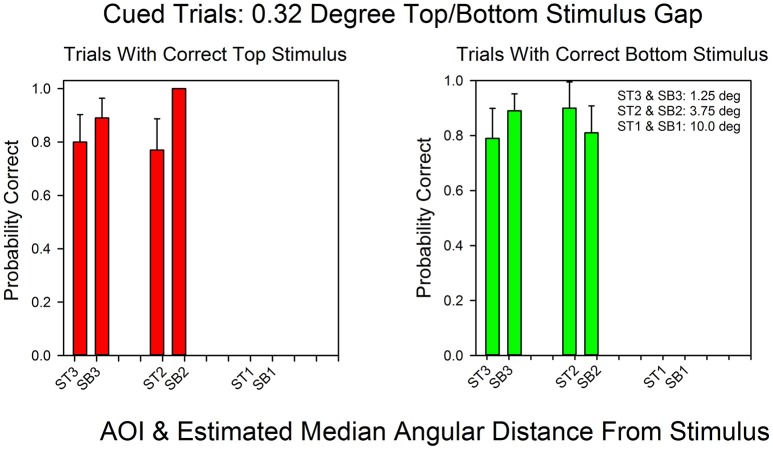
**Probability of a correct response as a function of AOI in cued trials in the 0.32° gap condition**. The format is the same as in Figure 3. The probabilities are based on the pooled results across all subjects and all correct row probabilities at the 0.32° stimulus gap setting.

Figure 4 shows the probability of a correct response as a function of AOI on cued trials in the standard condition (0.32° gap). In contrast to the 15° condition, the AOIs for correct top and bottom response were the same. The absolute likelihoods of a correct top and bottom responses were also similar, except at an angular distance of 3.75° (*ST2/SB2*). At this angular distance, the probability of a correct top stimulus response was 1.0, whereas the probability of a correct bottom stimulus response was 0.81. The Spearman rank correlation coefficient for the probabilities of correct top and bottom responses as a function of AOI was positive, as expected: *r*_*s*_ = 0.59, *p* < 0.11.

**Figure 5:**
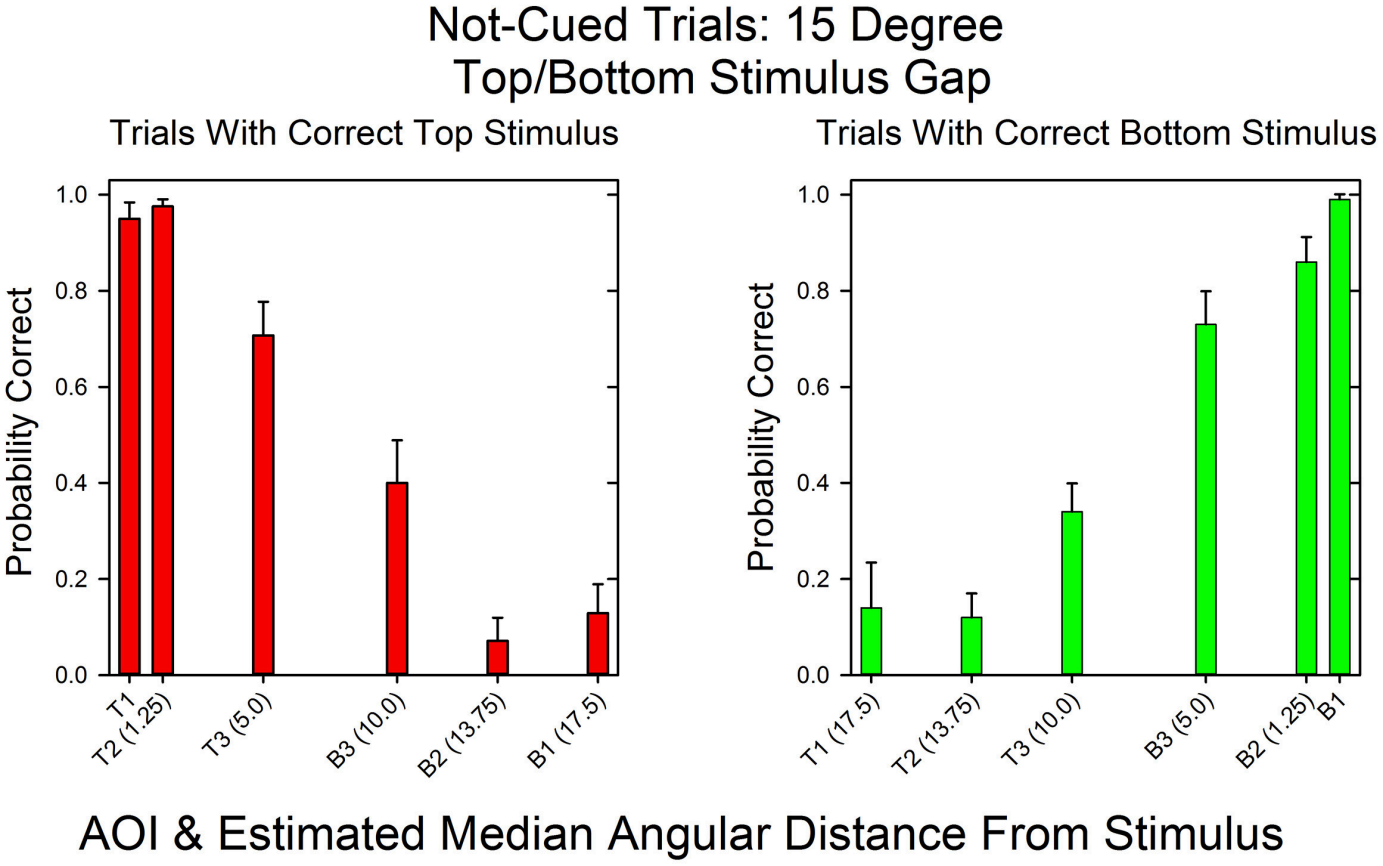
**Probability of a correct response as a function of AOI in not-cued trials in the 15°condition**. The format is the same as in Figure 3. The data are pooled across all subjects and all correct row probabilities in the 15°condition.

Figure 5 shows the relationship between AOI and probability of a correct response for not-cued trials in the 15°condition. As in the cued 15° condition, the distributions are mirror images. When the top stimulus had the matching sum, *T1* and *T2* had the highest probabilities of a correct response and *B2* and *B1* had the lowest probabilities of a correct response. In contrast, when the bottom stimulus had the matching sum, the AOIs with the highest and lowest probabilities were just the reverse. The Spearman rank correlation coefficient for the probabilities of correct top and bottom responses as a function of AOI was, as expected, negative: *r*_*s*_ = −0.94, *p* < 0.05. Also notice that in contrast to the cued condition, the subjects sometimes looked at distant AOIs in accordance with the uncertainty as to which stimulus would be correct.

**Figure 6:**
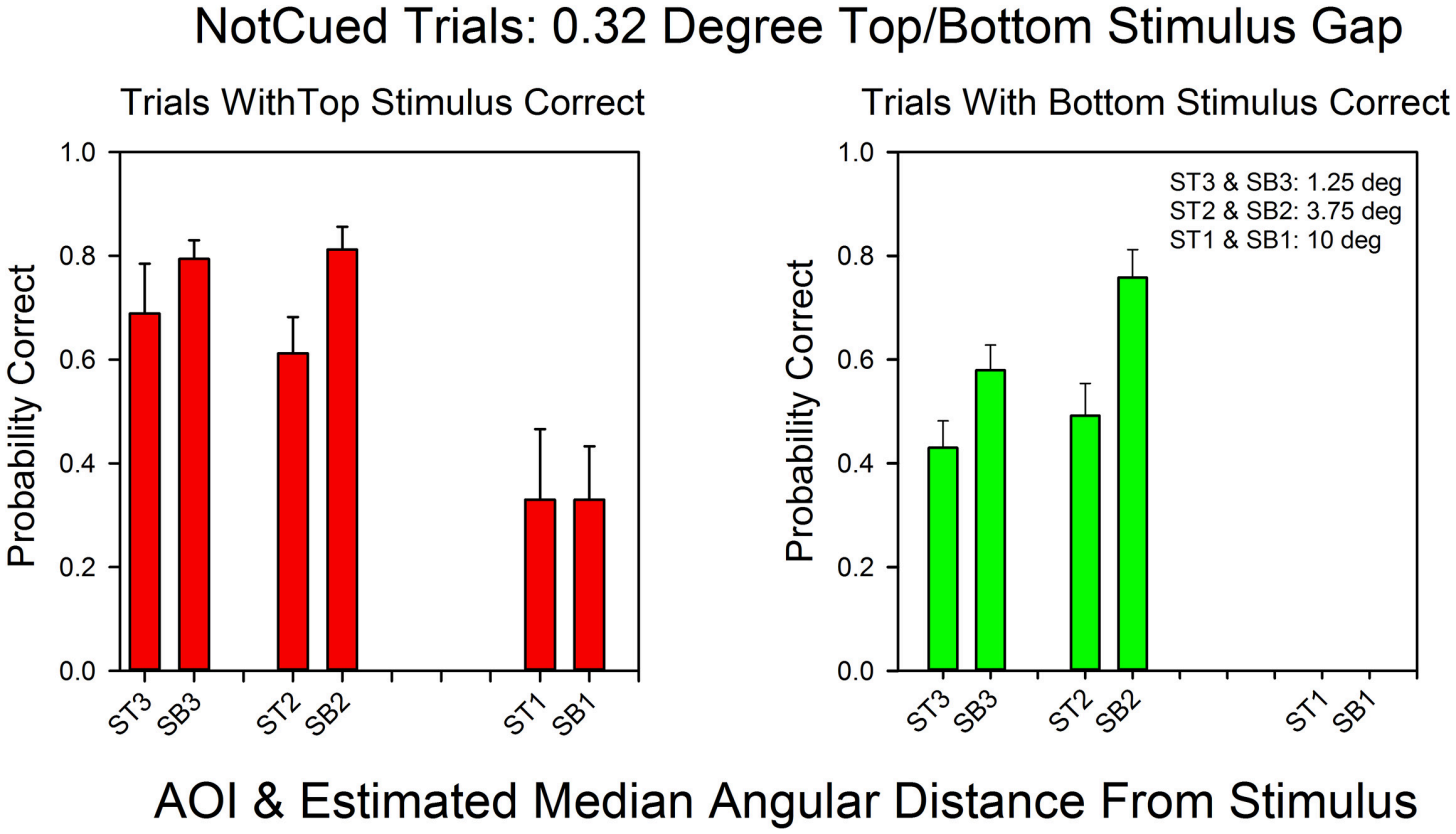
**The probability of a correct response as a function of AOI in not-cued trials in the 0.32°condition**. The format is the same as in Figure 3. The probabilities are based on the pooled results from all subjects and all correct row probabilities in the 0.32°condition.

Figure 6 shows the relationship between AOI and probability of a correct response for not-cued trials in the standard condition. Every AOI that supported correct bottom stimulus responses also supported correct top stimulus responses. However, the probability of a correct response was higher for the top stimulus at all AOIs, with an average difference of almost 30%, and subjects rarely focused on AOIs in the bottom half of the stimulus screen that were more than 3.75° from the center. As a result we were not able to calculate probabilities of a correct response on bottom correct trials for the 10° AOI. Nevertheless, when the AOIs are ranked according their probabilities of a correct response, the rankings were similar for top and bottom correct responses. The Spearman rank correlation coefficient was positive: *r*_*s*_ = 0.80, *p* < 0.10.

Table 1 summarizes the distribution of fixations for each of the four conditions of the study. The expected outcomes are that (1) on cued trials the distribution of fixations as a function of AOI should differ when the gap between the stimuli was large, but not when the gap was small, and that (2) the distributions should be less distinct in uncued trials, given the probabilistic nature of which stimulus was correct and that the subjects may have still been learning where to look and which stimulus to attend to. Put in terms of correlations, the distributions of fixations for top and bottom correct trials should be negatively correlated in the 15° condition, but positively correlated in the 0.32° condition. Table 1 supports both predictions. The Spearman rank correlations in the 15° condition were negative, as expected, with values of *r*_*s*_ = −0.93, *p* < 0.05 in cued trials and *r*_*s*_ = –0.37, *p* = 0.23 in not-cued trials. In the 0.32° (standard) condition, the Spearman rank correlations were positive as expected: *r*_*s*_ = 1.0, *p* < 0.05 in cued trials and *r*_*s*_ = 0.71, *p* = 0.06 in not-cued trials.

**Table 1:** **Percentage of fixations in an AOI as a function of experimental conditions**.

**AOI 15°**	**Top correct**		**Bottom correct**		**AOI 0.32°**	**Top correct**		**Bottom correct**	
	**Cued (%)**	**Not cued (%)**	**Cued (%)**	**Not cued (%)**		**Cued (%)**	**Not cued (%)**	**Cued (%)**	**Not cued (%)**
T1	37	16	0	5	ST1	0	3	3	3
T2	35	33	6	14	ST2	20	14	13	19
T3	23	16	2	22	ST3	23	8	20	27
B3	4	12	13	14	SB3	28	37	37	32
B2	0	11	30	16	SB2	25	22	23	18
B1	0	12	49	28	SB1	2	2	4	4

Recall that we did not calculate the probability of a correct response for trials in which there were fixations in two different AOIs (22% of trials). To check whether this might have affected the results, we also analyzed the data in terms of the cumulative amount of fixation time in an AOI, including trials in which there were fixations in two different AOIs. These results show the same patterns as Figures 3–6, except that on occasion the second fixation was in an AOI that was further from the target stimuli than in the single-fixation analyses. These fixations totaled 0.026 of the total fixation time.

**Figure 7:**
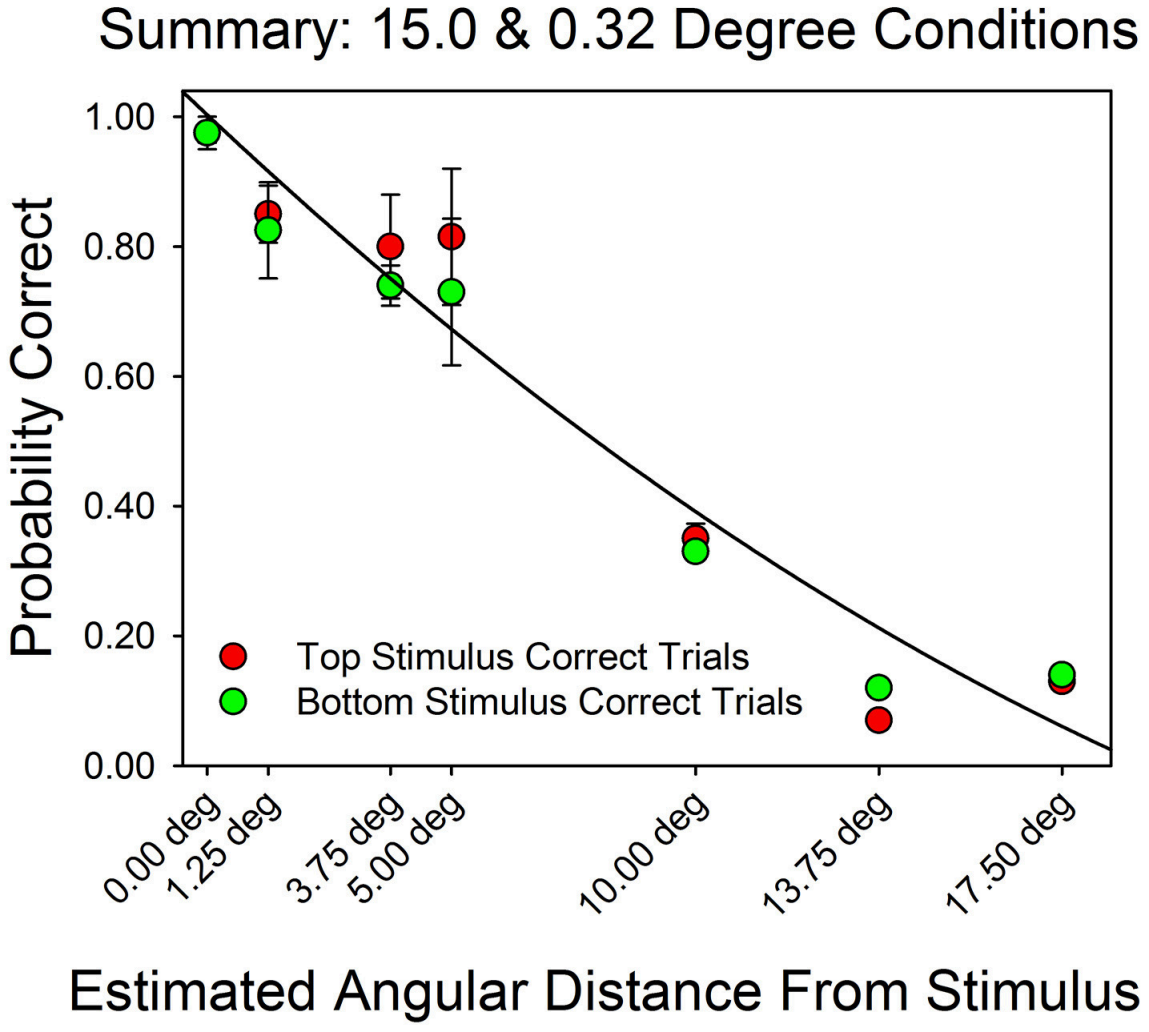
**Probability of a correct response as a function of estimated distance from the correct stimulus**. On the x-axis is the estimated midpoint angular distance from the correct stimulus. On the y-axis are the probability of a correct top row response (red filled circles) and the probability of a correct bottom row response (green filled circles). The line traces out the best fitting quadratic function. The error bars show standard errors. The graph is based on the pooled results from each subject in each condition.

Figure 7 summarizes the relationship between an AOI's distance from the target stimulus and probability of a correct response. On the x-axis is the AOI's median midpoint distance from the stimulus. On the y-axis is the probability of a correct response. Performance decreased as a function of angular distance, approximately linearly. However, the fitted line traces out a quadratic function (*R*^2^ = 0.95) since it better captures the fact that the probability of a correct response could not go to zero (because of the multiple choice format for responses). The fit for the linear function to these data has an *R*^2^ = 0.99). These results are consistent with the findings summarized by Figures 3–6.

**Figure 8:**
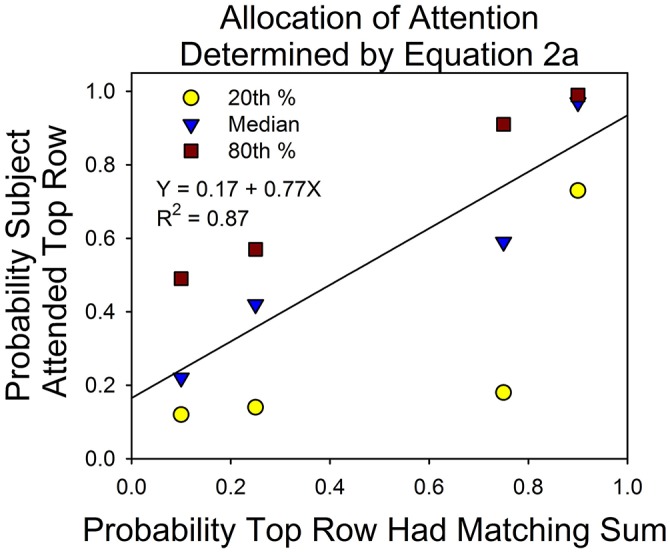
**Attention allocation according to Equation 2a**. On the x-axis is the probability that the top row had the matching sum in the probe screen. On the y-axis is the probability that the subject attended the top row, according to Equation 2. The data points are based on the results from the 0.32 and 15°conditions.

Figure 8 shows the division of attention between the two stimuli on uncued trials. This is the dependent measure of interest for understanding the principles that govern the allocation of attention. On the x-axis is the probability that the sum of three digits of the top stimulus matched one of the seven numbers in the probe screen. On the y-axis is the allocation of attention to the top stimulus according to Equation 2a. The data points are based on the 16 subjects. The line was fit to the median allocation results. Although there were only four subjects for each condition and only four data points, the median allocation values are consistent with the results from the first study in the conditions in which there was no monetary incentive or explicit feedback for correct answers (see the *Discussion* section for details).

Table 2 lists performance parameters relevant to the allocation results: the duration of the stimulus screen, as determined by the calibration procedure, the probability of a correct response on trials in which the subject was told beforehand which stimulus was correct (which is the parameter *A* in Equations 2a and 2b), and the probability of a correct guess (*g*), as determined by Equation 2b. The median probability of a correct guess was 0.161, which was not significantly different from the expected value of 0.143 according to a Wilcoxon Signed-Ranks test (*Z* = −0.931, *p* = 0.35). This test was based on the square root transformed values of 0.143 and *g* (because many values were close to 0.0).

**Table 2:** **Stimulus exposure time, accuracy, correct guess rate**.

**Percentile**	**Exposure time (ms)**	**Accuracy top stimulus**	**Accuracy bottom stimulus**	**Correct guess rate**
20th	135.0	0.91	0.79	0.077
50th	157.5	0.91	0.91	0.161
80th	180.0	1.00	1.00	0.320

## Discussion

We will address the following issues: whether correct answers in the standard procedure (0.32° gap) depended on covert shifts in attention, whether the probabilities of attending the top and bottom row stimuli approximated the probabilities that these stimuli had a matching sum in the probe screen, the size of the effective field of view, and evidence that the principles that govern the allocation of choice in behavioral experiments also govern the allocation of attention in cognitive experiments.

The following logic and results demonstrate that shifts of attention rather than shifts in eye movements mediated correct responses in the standard procedure. Figures 4 and 6 show the relationship between AOI and correct responses over the course of the session. The graphs demonstrate that the same fixation, as measured by AOI, supported correct responses at both the top and bottom stimulus. However, on any given trial, correct responses were limited to either the top stimulus or the bottom stimulus. For instance, according to the Wilcoxon Signed-Ranks test, the probability that a subject correctly identified the sum of the three digits at the unattended stimulus did not differ from chance. Together these findings imply trial-to-trial shifts in attention, and since fixations did not shift, covert attention must have shifted.

Figure 8 shows that shifts in attention were correlated with the probabilities of a correct response. On the x-axis is the probability of a correct response at the top stimulus; on the y-axis is the probability that the subjects attended the top stimulus. The slope of the best fitting line to the median allocation values was 0.77. This is well short of a slope of 1.0 for perfect probability matching, but very close to the expected result on the basis of the previous experiment. To see this, some calculations are necessary.

On the basis of our initial study, we estimated the expected parameters for the line that would best describe the correlation between the programmed probabilities of a correct answer and probabilities that the subjects attended the top and bottom rows (as in Figure 8). For the condition that was most similar to the present study, the intercepts and slopes for the best fitting lines were 0.35 and 0.48 for the first session and 0.17 and 0.70 for the second session. Figure 8 summarizes the allocation results for a mixture of five first and eleven second eye-tracking sessions. Combining these observations (and assuming that the first study provides a perfect guide to the present study), we obtained an expected intercept and slope of 0.23 and 0.64, respectively. For example, the expected intercept is [(5 × 0.35) + (11 × 0.17)]/16 = 0.23. The observed slope and intercept were 0.17 + 0.77. The difference implies that attention allocation was more strongly correlated with the probabilities of a correct response in the present study. This may reflect differences in the rate of learning in the two experiments. For instance, when we calculated the expected intercept and slope based on the 2nd half of each session from the previous study, the result closely approximated what was observed in this study: an expected intercept of 0.15 and an expected slope of 0.78—an almost perfect match to the results of this study.

Figure 7 provides an estimate of what is referred to as the “effective” or “functional field of view.” This is the domain, measured in degrees, over which a stimulus can attract attention. Research shows that it is wider than the fovea's domain and is strongly influenced by individual differences, such as age, and perceptual factors, such as the shape and arrangement of the stimuli, and semantic factors, such as whether the stimulus array includes a dangerous object, such as a weapon (Ball et al., 1988; Williams, 1989; Harada et al., 2015). Figure 7 shows that for distances of about 1.25 to 5.0°, performance remained at about 80% correct. This finding is consistent with earlier studies on the effective field of view subjects of about the same age as ours (e.g., Ball et al., 1988; Williams, 1989). In these experiments, college subjects were able to effectively extract information from stimuli that were up to 5.0° apart (e.g., Williams, 1989).

## Limitations

The method of measuring angular distance from an AOI to the target stimuli was approximate. The AOIs differed in area, and we used their midpoint as a summary distance. The actual average points of regard must have differed at least somewhat from the nominal measures. However, if the actual points of regard were distributed in similar ways for each AOI then the median midpoint distance should prove a reasonably accurate approximation of the actual points of regard. In support of this reasoning, the best fitting quadratic equation accounted for 95% of the variance in the relationship between angular distance and accuracy, and the best-fitting linear equation accounted for 99% of the variance in the distance/accuracy pairs.

The correlations were limited by the constrained range of the fixations in the 0.32° degree condition. The subjects learned that fixations in the middle of the screen would allow them to correctly respond to both the top and bottom stimuli so that there were no more than four pairs of values. As a result the correlations were not significant at the 0.05 level despite the fact that the correlations were high (e.g., 0.59 and 0.80). However, our goal was to test the validity of calculations based on the size of the fovea and the stimuli. These calculations implied that our procedure and equation measured the allocation of covert attention. As emphasized, Figures 4, 6 strongly support our approach. If Equations 2a and 2b correctly calculate attention allocation then these two graphs should reveal that the AOIs for top and bottom correct responses should overlap in the standard condition and not overlap in the 15° condition. The figures show that this is precisely what happened. Hence, it is not clear what a lower *p*-value would add to our understanding of the results.

This study and its predecessor are silent in regards to the details of the psychological processes that mediated the limits in processing the stimuli and their underlying neural mechanisms. These limitations may include constraints in encoding the digits in each row and/or constraints on working memory. However, our initial goal is to test whether the allocation of cognitive capacities in an attention procedure follow the same quantitative principles as the allocation of overt behavior in choice procedures. If the results continue to support this approach, it will be possible to then test the nature of the constraints at work in this study. The procedure is simple and quantitative so that it will lend itself to experiments that attempt to unpack the cognitive processes at work in matching the digits in the stimulus screen to their sum in the probe screen.

## Summary

Figures 3, 6 and Tables 1, 2 support the idea that Equation 1 provides valid quantitative estimates of the allocation of attention. The results also demonstrate that the participants shifted attention without shifting their gaze. In the behavioral version of the procedure used in this study (“two-armed bandit” experiments), response proportions approximate the probabilities of a correct response when there is no feedback but shift toward maximizing as a function of feedback, practice and incentives. In this study there was no feedback. As predicted by the behavioral studies, the allocation of attention more closely approximated probability matching than maximizing. This replicates the results of the previous study using the same procedure. Thus, the results support the hypothesis that the principles that govern the allocation of choice also govern the allocation of attention.

## Ethics statement

This study was carried out in accordance with the recommendations of the Boston College IRB with written informed consent from all subjects. All subjects gave written informed consent in accordance with the Declaration of Helsinki. The protocol was approved by the Boston College IRB.

## Author contributions

GH helped design the study, analyze the results and write the manuscript. JM helped design the study and analyze the results. KG helped run the experiments and write the manuscript.

## Funding

The research was generously funded by Boston College's Ignite Program.

### Conflict of interest statement

The authors declare that the research was conducted in the absence of any commercial or financial relationships that could be construed as a potential conflict of interest.
